# Polyamines in Pollen: From Microsporogenesis to Fertilization

**DOI:** 10.3389/fpls.2016.00155

**Published:** 2016-02-18

**Authors:** Iris Aloisi, Giampiero Cai, Donatella Serafini-Fracassini, Stefano Del Duca

**Affiliations:** ^1^Dipartimento di Scienze Biologiche, Geologiche e Ambientali, Università degli Studi di BolognaBologna, Italia; ^2^Dipartimento di Scienze della Vita, Università di SienaSiena, Italia

**Keywords:** fertilization, microsporogenesis, polyamines, putrescine, self-incompatibility, spermidine, spermine, transglutaminase

## Abstract

The entire pollen life span is driven by polyamine (PA) homeostasis, achieved through fine regulation of their biosynthesis, oxidation, conjugation, compartmentalization, uptake, and release. The critical role of PAs, from microsporogenesis to pollen–pistil interaction during fertilization, is suggested by high and dynamic transcript levels of PA biosynthetic genes, as well as by the activities of the corresponding enzymes. Moreover, exogenous supply of PAs strongly affects pollen maturation and pollen tube elongation. A reduction of endogenous free PAs impacts pollen viability both in the early stages of pollen development and during fertilization. A number of studies have demonstrated that PAs largely function by modulating transcription, by structuring pollen cell wall, by modulating protein (mainly cytoskeletal) assembly as well as by modulating the level of reactive oxygen species. Both free low-molecular weight aliphatic PAs, and PAs conjugated to proteins and hydroxyl-cinnamic acids take part in these complex processes. Here, we review both historical and recent evidence regarding molecular events underlying the role of PAs during pollen development. In the concluding remarks, the outstanding issues and directions for future research that will further clarify our understanding of PA involvement during pollen life are outlined.

## Forms, Molecular Partners, and Tasks of Polyamines

**FIGURE 1:**
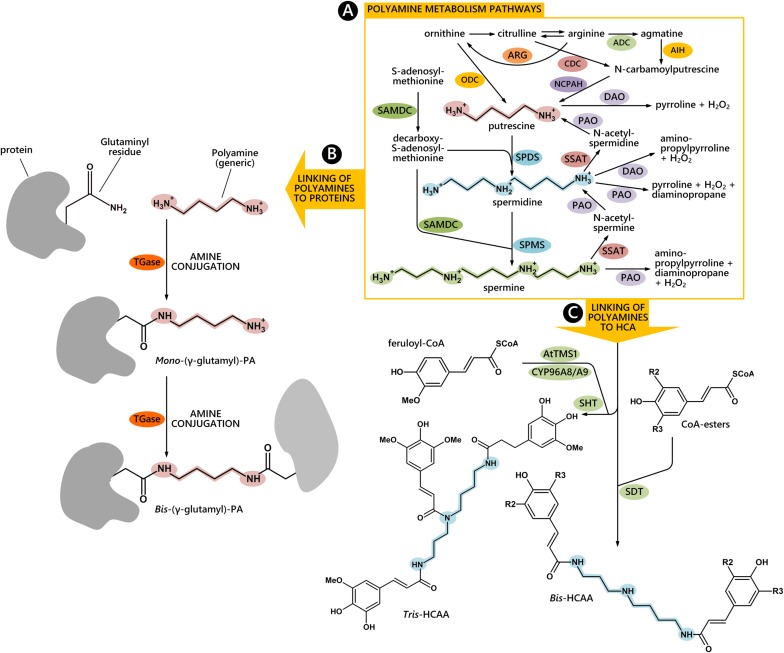
**PAs metabolism and their conjugating pathways to proteins and to hydroxyl-cinnamic acids (HCA).** Free PA biosynthetic and catabolic pathways are highlighted in the yellow rectangle **(A)**. The covalent binding to glutamyl residues of proteins gives rise to mono-γ glutamyl-PAs or to cross-links between proteins (bis-γ glutamyl-PAs) **(B)**. The biosynthetic pathway of hydroxyl-cinnamic acids amides (HCAAs) in *Arabidopsis thaliana* stamens is reported according to Fellenberg et al. (2012) **(C)**. ADC, arginine decarboxylase; ARG, arginase; AIH, agmatine iminohydrolase; CDC, citrulline decarboxylase; NCPAH, *N*-carbamoylputrescine amidohydrolase; ODC, ornithine decarboxylase; SAMDC, *S*-adenosylmethionine decarboxylase; SPDS, spermidine synthase; SPMS, spermine synthase; PAO, polyamine oxidase; SSAT, spermidine/spermine *N*^1^-acetyltransferase; DAO, diamine oxidase; TGase, transglutaminase; SHT, Spd hydroxycinnamoyl transferase; CYP98A8/CYP98A9, P450 cytochromes; AtTMS1, *Arabidopsis thaliana* tapetum-specific methyltransferase, SDT, spermidine disinapoyltransferase.

In plant cells, metabolism of aliphatic PAs occurs in the cytosol and organelles (**Figure 1A**); Put has an aliphatic tetramethylene backbone deriving directly from ornithine or indirectly from arginine or citrulline via *N*-carbamoylputrescine. The biosynthesis of higher PAs occurs by the addition of one or two aminopropyl groups to Put to form Spd and Spm, respectively. Whereas Put has positive charges on the primary amino groups, Spd and Spm also bear protonated internal iminic groups, at physiological pH. PAs are present in cells in both free and bound forms and their molecular mechanism of action is often associated with their polycationic groups able to establish hydrogen and ionic interactions with anionic groups of several biological molecules, among which proteins, nucleic acids, and membrane phospholipids. Moreover, they strongly bind *in vitro* to cell wall polysaccharides with a different binding capacity depending mainly upon the number of their positive charges. In addition, the covalent binding to some glutamyl residues of specific proteins, catalyzed by TGase, gives rise either to PA binding to proteins (*mono*-γ glutamyl-PAs) or to cross-links between proteins (*bis*-γ glutamyl-PAs) (**Figure 1B**). These conjugates are components of the PCA-insoluble PA fraction (Del Duca et al., 2014). Covalent binding of PAs to phenylpropanoids, such as HCA, abundant in many plant families, give rise to hydroxyl-cinnamic acids amides (HCAAs) (**Figure 1C**), components of the PCA-soluble fractions. These are involved in the organization of the cell wall and are associated to fertility (Martin-Tanguy, 2001; Grienenberger et al., 2009).

In plant cells, PAs are mostly stored in the vacuole and in the cell wall, but Spm is present also in the nucleus (Belda-Palazon et al., 2012). PAs play a molecular stabilizing role by crossing the DNA double helix and covalently binding to histones, thus controlling transcription. Moreover, PAs are believed to act as radical scavengers thereby protecting DNA from ROS (Das and Misra, 2004). During catabolism, PAs and in particular Spm, are suggested as a source of free radicals (Takahashi and Kakehi, 2010). The role of PAs in plant cell life, therefore, appears multifaceted; in some instances, they act as pro-survival molecules, whereas in others they accelerate PCD (Cai et al., 2015a). Indeed, it is not astonishing that the perturbation of PA homeostasis influences many fundamental cell processes (Tiburcio et al., 2014), such as organogenesis, cell proliferation, differentiation, senescence/PCD, and stress- and external stimuli-induced homeostatic adjustments. Special issues on PAs have been reported (http://www.frontiersin.org/books/Plant_polyamines_in_stress_and_development/340 and http://www.sciencedirect.com/science/journal/09819428/48/7).

Polyamines also control many aspects of pollen development, both under normal and stress conditions. Here, we summarize the involvement of PAs during the entire developmental program and functioning of pollen.

## Polyamines in Pollen

### Microsporogenesis

**FIGURE 2:**
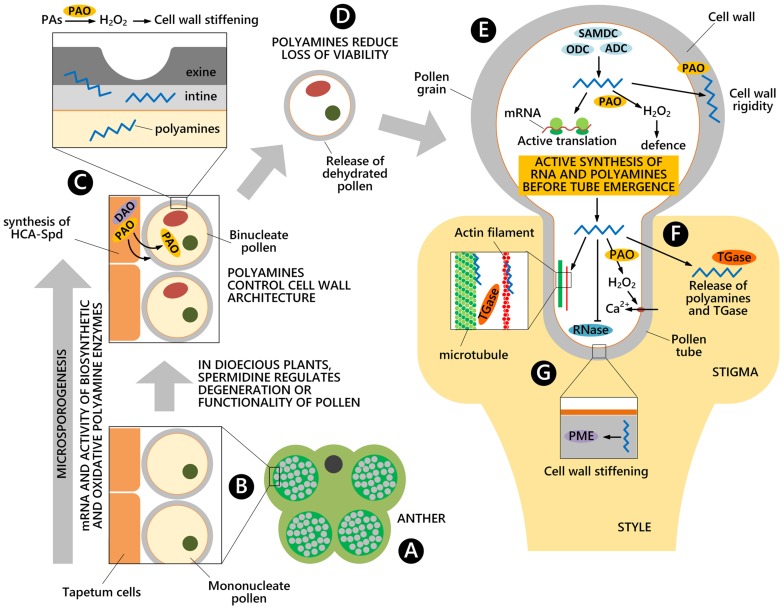
**Polyamines involvement during pollen development.** PA biosynthetic and oxidative metabolisms occur from the early stage of pollen formation inside the anthers **(A)**, when both microspores and the tapetal cell layer of the anther contribute to microspore cell wall architecture **(B)**. Pollen accumulates high levels of free PAs and HCAAs, mainly localized in the cell wall. PA catabolism by PAO and DAO modulates the rigidity of the cell wall **(C)**. Once dehydrated, pollen grains are released and PAs contribute to maintain pollen viability **(D)**. During germination on a stigma **(E)**, PAs promote the translation of transcripts and they are also released in the extracellular space, together with TGase **(F)**. During pollen tube growth in a compatible style, PAs take part in the cytoskeleton organization, in cell wall deposition and remodeling by the PME enzyme as well as in the regulation of ion transport through the plasma membrane. PAs also exert an inhibitory effect on RNase enzymes **(G)**.

Transcripts for enzymes involved in PA biosynthetic and oxidative metabolisms are present starting from the early pollen stages as observed during *Nicotiana tabacum* pollen formation inside the anthers (**Figure 2A**). At the stage of uninucleate microspore, transcripts for enzymes involved in the biosynthesis of PAs, mostly Put, have been found, namely transcripts for ADC and ODC (Bokvaj et al., 2015) (**Figure 2B**). At the bicellular pollen stages, other transcripts are present for the oxidative metabolism of Put (e.g., DAO) (**Figures 1A and 2C**); additional transcripts for enzymes that participate in the urea cycle and metabolism of amino groups (e.g., *N*-carbamoylputrescine amidase) are also present (**Figure 1A**).

Both the sporophytic tapetal layer of the anther and the gametophyte contribute to the formation of the pollen grain cell wall, consisting of the inner intine and the outer exine layers. This process is not only strictly related to the deposition of cell wall components necessary for fertilization and protection against biotic and abiotic stresses, but is also essential for enzymatic reactions. When present, tryphine, the soluble part of the pollen exine, is the preferential accumulation site of soluble HCAAs. Recent studies in *Arabidopsis thaliana* demonstrated that HCAAs are exported from the tapetum prior to dehiscence of the anthers, which occurs by PCD (Quilichini et al., 2014). HCAAs form a highly variable mixture, made of at least 30 different (HCA)-Spd conjugates (Handrick et al., 2010) (**Figure 1C**). These compounds were shown to crosslink different cell wall polymers via ester and ether linkages, suggesting a role in modulating the rigidity of the cell wall (Moschou et al., 2012). The enzyme SHT (**Figure 1C**), catalyzing the conjugation of hydroxycinnamoyl CoA to Spd in anthers, was recently shown to take part in the organization of the cell wall. The *sht* mutant displayed irregularities, depressions and decreased auto-fluorescence of the pollen grain (Grienenberger et al., 2009). It also displayed disappearance of tris-HCAAs from Spd conjugates, whereas the qualitative and quantitative pattern of bis-HCAAs was much less affected (Handrick et al., 2010). These conjugates have been found sporadically in other species but their role remains to be established (Fellenberg and Vogt, 2015). Elejalde-Palmett et al. (2015) showed that an acyltransferase of *Malus domestica* was able to complement the *sht* mutant of *Arabidopsis thaliana.* Based on bioinformatic analyses of putative SHT orthologs, authors showed a genetic linkage among *SHT* sequences and argued for a common ancestral origin of the *SHT* gene in a common core Eudicotyledon ancestor (Elejalde-Palmett et al., 2015). Recently, a second transferase, Spd disinapoyl transferase (SDT), was shown to be considerably expressed in stamens and involved in the formation of HCAAs (Fellenberg et al., 2012). In addition to the reaction catalyzed by SHT/SDT, at least two subsequent reactions that add phenolic rings were shown to be catalyzed by tapetum-specific CYP98A8/CYP98A9 (Matsuno et al., 2009) and an AtTMS1 (Fellenberg et al., 2008) (**Figure 1C**). Recently, the biosynthetic pathway of (HCA)-Spd based on the analysis of several *Arabidopsis* knock-out mutants was proposed (Fellenberg et al., 2009). PAs were thus shown to contribute directly to wall architecture. It was, however, proposed that they also control wall stiffening indirectly by regulating PME (**Figure 2G**) (Charnay et al., 1992).

When oxidized by PAO, PAs may play an additional role during pollen development in so far as the reaction product H_2_O_2_ is involved in cell wall stiffening. Pollen PAOs (Wu et al., 2010; Fincato et al., 2012), but also apoplastic PAOs secreted from the anther, appear to be involved (**Figure 2C**). In *Oryza sativa* seven PAO isoforms have been identified, and one of these, OsPAO7, is specifically expressed in anthers, with an expression peak at the bicellular pollen stage (**Figure 2C**); OsPAO7 produces H_2_O_2_ about 100 times more efficiently than other PAO isoforms (Cona et al., 2006; Liu et al., 2014).

In the dioecious kiwifruit, Put and Spd represent biochemical markers for male sterility in female plants by being involved in female pollen degeneration. During microgametogenesis, ADC, ODC, and SAMDC, the latter involved in Spd/Spm biosynthesis (**Figure 1A**) are active. The aborted pollen grains showed high SAMDC activity in wall residues, while functional pollen (from the male-fertile anthers) showed low SAMDC activity, suggesting a possible regulatory role of Spd in the functionality of kiwifruit pollen (Falasca et al., 2010). The involvement of tapetal SAMDC in pollen development and male fertility was also demonstrated in tomato by RNAi techniques. Down-regulation of several tapetal SAMDC homologs not only led to reduction in cellular PA levels, particularly in the bound and conjugated forms, but also caused partial or complete male sterility in transgenic plants. RNAi-mediated downregulated *SAMDC* lines showed morphological abnormalities only in the pollen grains, which were shrunken and distorted (Sinha and Rajam, 2013).

### Quiescence and Viability

Pollen can be stored for extended periods without loss of viability under dry and low-temperature conditions leading to reduced metabolism. PAs may contribute to maintaining viability during natural quiescence and/or storage (**Figure 2D**), when the main PA biosynthetic enzymes (i.e., ADC, ODC and SAMDC) were present and active *in vitro* (Falasca et al., 2010). Two different *SAMDC* gene transcripts were highly expressed together with weak *ADC* transcription. The combined application *in planta* of competitive inhibitors of SAMDC (methylglyoxal-bis guanylhydrazone) and Spd synthase (SPDS) (cyclohexylamine), or D-arginine (inhibitor of Put synthesis) led to abnormal pollen grains in male-fertile plants with reduced viability and germination (Falasca et al., 2010). Reduced pollen viability was associated to a lower activity of the PA biosynthetic enzymes upon rehydration; in fact, exogenous PAs applied to germination medium were able to restore germination and fertilization of aged pollen grains (Song and Tachibana, 2007) (**Figure 2D**).

### Pollen Rehydration and Pollen Tube Emergence

Different RNAs and proteins are synthesized at the onset of pollen germination (Linskens et al., 1968; Bagni et al., 1981). Spd was shown to play a role in male gametophyte development of *Marsilea vestita*, a heterosporous fern, by unmasking the translationally inhibited stored mRNAs (Deeb et al., 2010; Boothby et al., 2013). Spd was hypothesized, but not demonstrated, to play a similar role in pollen of flowering plants. It is noteworthy that inhibition of pollen germination by the transcriptional inhibitor actinomycin D (Speranza et al., 1986) or by the protein synthesis inhibitor cycloheximide could be overcome by treatment with exogenous Spd and Spm (Song and Tachibana, 2007). High activities of PA biosynthetic enzymes, in particular during the very early stages of germination, were detected in different pollens (Bagni et al., 1981; Falasca et al., 2010) (**Figure 2E**). Moreover, the inhibition of PA biosynthetic enzymes by bis(guanylhydrazones) strongly affected pollen germination (Antognoni and Bagni, 2008).

Despite high biosynthetic enzyme activities, the amount of both free and bound Spd was shown to decrease concomitantly. The PA was released into the germination medium together with RNAs, neo-synthesized proteins (Bagni et al., 1986), and TGase, suggesting their possible involvement in pollen tube/style adhesion (Di Sandro et al., 2010). In general, profiles of PAs, RNAs, and proteins during germination seem to be finely co-regulated.

As PA homeostasis must be finely tuned, exogenous application of PAs has dramatic effects on pollen germination. Low concentrations of exogenous PAs were often shown to stimulate pollen tube emergence while high concentrations drastically altered tube growth and morphology (Antognoni and Bagni, 2008; Wu et al., 2010; Rodriguez-Enriquez et al., 2013; Aloisi et al., 2015). It was suggested that Spd could increase *in vitro* pollen germination by reducing local effects of pollen density, which negatively affects this process (Rodriguez-Enriquez et al., 2013).

Interestingly, both RNA and protein biosynthesis (Bagni et al., 1981) were shown to be stimulated by addition of Spd, but were inhibited by an excess of Spm, as first observed in *Petunia* (Linskens et al., 1968). Because PAs (which can also be RNA bound) promote both transcription and translation, a positive feedback could be hypothesized (Bagni et al., 1973, 1986). It has been proposed that Spd and Put may play a role in the developmental change from monosomes to polysomes, the process needed for active protein synthesis during pollen tube germination (Falasca et al., 2010).

### Pollen Tube Growth

A strict regulation of the influx/efflux of inorganic ions (mostly Ca^2+^ and K^+^) across the plasma membranes, the apical pool of ROS (Potocky et al., 2007) and a highly dynamic and polarized cytoskeleton ensure polarized growth at the pollen tube apex. In *Rosaceae*, the effect of exogenous PAs during pollen tube growth seems multifactorial and was shown to involve the organization and assembly of the cytoskeleton (Del Duca et al., 2009) and cell wall deposition (Di Sandro et al., 2010). The action of PAs is at least in part mediated by TGase that is present in distinct cell sites, including cytosol, organelles, membranes and cell walls, all involved in PA metabolism. TGase was reported to mediate pollen germination and pollen–style interactions (Del Duca et al., 2013) (**Figures 2F,G**). In fact, during pollen tube growth, the activity of cytoplasmic TGase was mainly detected in the tube apex and in the region closest to the grain. PA conjugation to actin and tubulin, catalyzed by TGase, affected their ability to assemble and their interaction with motor proteins both *in vivo* and *in vitro* (Del Duca et al., 2009). TGase, co-localizing with pectins and arabinogalactan-proteins in the cell wall, was released during tube elongation (Del Duca et al., 2013). This extra-cellular TGase and its products localized as aggregates at the surface of *Malus domestica* pollen tubes. As specific TGase inhibitors blocked tube growth, a role for TGase in tip growth and in the reinforcement of the cell wall, supporting the migration of pollen tubes through the style, was proposed (Del Duca et al., 2013) (**Figures 2F,G**). Moreover, pollen TGase secreted into the medium catalyzed the covalent linkage of PAs to released proteins and their cross-linking *in vitro.* This feature may contribute to regulating the pollen tube-style interaction (Di Sandro et al., 2010).

In addition, PAs might also control the assembly and properties of cell wall polysaccharides, such as pectins, which bind to PAs by ionic linkages (D’Orazi and Bagni, 1987). In cell walls of soybean, positively charged PAs competed with acidic pectins in binding calcium ions; moreover, PAs were reported to regulate the activity of PME, thereby leading to decreased levels of acidic pectins and, therefore, to softer cell walls (Charnay et al., 1992) (**Figure 2G**).

In *Arabidopsis thaliana* pollen tubes, exogenously supplied Spd increased the concentration of cytosolic Ca^2+^; Spd oxidation by PAO generated H_2_O_2_, which activated Ca^2+^ channels, thus inducing Ca^2+^ influx beyond optimal levels and causing the inhibition of tube growth. Activation of Ca^2+^ currents by Spd was significantly disrupted in *pao* knock-out mutants, but Ca^2+^ channels could still be activated following application of H_2_O_2_ (Wu et al., 2010).

Spm was the most effective PA in inhibiting pear pollen tube elongation (Aloisi et al., 2015). Spm rapidly entered the pollen tube tip and caused swelling of the apex, suggesting cell wall relaxation. Spm rapidly induced ROS formation (Pottosin et al., 2014; Aloisi et al., 2015), causing the reduction of pollen viability, followed by activation of the antioxidant machinery. The final event after Spm supply was the degradation of nuclear DNA leading to cell death; this process was proposed to be induced either by Ca^2+^-activated signaling or by the altered redox state (Aloisi et al., 2015).

### Pollen–Pistil Interaction During Fertilization and Self-Incompatibility

When pollens land on an incompatible stigma they may undergo the Self Incompatibility (SI) response. This is the most important evolutionary system of the Angiosperms to prevent inbreeding and requires a species-specific cell–cell recognition system. The female determinants can be either a cell membrane receptor as in *Papaver rhoeas* or a released molecule, such as stigma/style ribonucleases (termed S-RNases) in *Solanaceae*, *Rosaceae* and *Plantaginaceae*; they enter the pollen and are degraded in compatible pollen while they are active in incompatible ones causing the degradation of pollen RNA (Dresselhaus and Franklin-Tong, 2013).

The involvement of PAs in the SI response has been reported both in *Pyrus communis* and in *Citrus grandis*. In *Pyrus communis* the content of free PAs (Put and Spm) was lower during incompatible as compared to compatible pollination (**Figures 2F,G**). This could be related to the inhibitory effect of PAs on RNases; in fact, Put and Spd, and, even more, Spm, have been shown to halve the activity of RNase in *Malus domestica* pollen (Speranza et al., 1984), as also observed in *Solanum tuberosum* (Altman, 1982).

The accumulation of PCA-soluble PAs in reproductive organs, and particularly in pollen, has been associated with fertility. Triferuloyl-Spd, a HCAA of tryphine, is involved in pollination and in pollen–stigma interaction. Moreover, the amount of PCA-soluble PAs was lower in SI-pollinated styles compared to compatible pollinated ones. In the SI-pollination styles, an increase of PCA-insoluble PAs and a higher TGase activity were also observed, concomitantly with the arrest of tube growth and the appearance of a TGase plug at the tip (Del Duca et al., 2010).

In contrast to compatible pollination, SI pollination in *Citrus grandi*s was characterized by higher amounts of PCA-insoluble PAs, enhanced TGase activity, and increased production of glutamyl-PAs, together with arrested pollen tube growth (Gentile et al., 2012). The direct involvement of the cytoskeleton in SI was so far solely reported in incompatible *Papaver* tubes, where a high Ca^2+^ influx took place after pollen–stigma interaction. Subsequently, F-actin foci were formed by a still uncharacterized cross-linking mechanism, leading to the arrest of tube elongation and to pollen PCD (McClure and Franklin-Tong, 2006). Since enhanced Ca^2+^ influx is a general feature of the SI response, this could account for the fact that activity of TGase (which is a Ca^2+^-dependent enzyme) was stimulated in *Pyrus communis* and *Citrus grandis.* This could have led to cross-links among cytoskeleton proteins, generating high-mass aggregates, similar to the actin foci observed in *Papaver*, and forming the tube tip plug (Del Duca et al., 2014; Cai et al., 2015b).

## Conclusion

Pollen development is a complex and well-coordinated process governed by genetic and enzymatic processes, some of which are modulated by PAs. Hence, these aliphatic polycations, drive pollen development throughout its life span, as summarized in **Figure 2.** Progress in past decades has significantly advanced our understanding of how PAs exert multiple roles by different molecular mechanisms. However, further investigations on the physiological function of PAs and their molecular partners are still needed. In particular, knowledge would strongly benefit from a deeper understanding of PA transporters, which have been poorly studied. This could provide new insights on the interactions between the tapetal layer and the pollen grain during its development in the anther. It could likewise explain how HCAAs, PA biosynthetic and oxidative enzymes and other cell wall components are deposited during microsporogenesis. Moreover, despite recent findings on the composition and biosynthetic pathway of pollen HCAAs, clear evidence regarding their functions is still lacking. While some of the roles of PAs are rather evident, e.g., modulation of the cytoskeleton by TGase, others remain elusive, e.g., PA interactions with nucleic acids. Such information could explain their possible role in epigenetic control, the interconnection between PAs and ROS, and the role of free and conjugated PAs in the apoplast during the pollen–pistil interaction.

## Author Contributions

IA, GC, DS-F, SD contributed to the design of the work as well as drafting the work and revising it critically for important intellectual content; then they made the final approval of the version to be published. They agree to be accountable for all aspects of the work in ensuring that questions related to the accuracy or integrity of any part of the work are appropriately investigated and resolved. In details, the idea to write a paper about the polyamines action during pollen life was proposed by IA and SD that is the supervisor of the entire work. Part of the data described in the text have been done in the labs of Bologna and Siena both with a solid expertise in polyamine and pollen. GC designed and realized the figure of the paper.

## Conflict of Interest Statement

The authors declare that the research was conducted in the absence of any commercial or financial relationships that could be construed as a potential conflict of interest.

## Abbreviations

ADC: arginine decarboxylase
AIH: agmatine iminohydrolase
ARG: arginase
AtTMS1: *Arabidopsis thaliana* tapetum-specific methyltransferase
CDC: citrulline decarboxylase
CYP98A8/CYP98A9: P450 cytochromes
DAO: diamine oxidase
HCA: hydroxyl-cinnamic acids
HCAA: hydroxycinnamic acids amide
NCPAH: *N*-carbamoylputrescine amidohydrolase
ODC: ornithine decarboxylase
PAO: polyamine oxidase
PAs: polyamines
PCA: perchloric acid
PCD: programmed cell death
PME: pectin methyl-esterase enzymes
Put: putrescine
ROS: reactive oxygen species
S-RNase: locus *S*-ribonuclease
SAMDC: S-adenosylmethionine decarboxylase
SDT: spermidine disinapoyltransferase
SHT: Spd hydroxycinnamoyl transferase
SI: self-incompatibility
Spd: spermidine
SPDS: spermidine synthase
Spm: spermine
SPMS: spermine synthase
SSAT: spermidine/spermine *N*^1^-acetyltransferase
TGase: transglutaminase
